# An automatic device for detection and classification of malaria parasite species in thick blood film

**DOI:** 10.1186/1471-2105-13-S17-S18

**Published:** 2012-12-07

**Authors:** Saowaluck Kaewkamnerd, Chairat Uthaipibull, Apichart Intarapanich, Montri Pannarut, Sastra Chaotheing, Sissades Tongsima

**Affiliations:** 1 National Electronics and Computer Technology Center (NECTEC), National Science and Technology Development Agency (NSTDA), Thailand Science Park, Pathumthani 12120, Thailand; 2 National Center for Genetic Engineering and Biotechnology (BIOTEC), National Science and Technology Development Agency (NSTDA), Thailand Science Park, Pathumthani 12120, Thailand

## Abstract

**Background:**

Current malaria diagnosis relies primarily on microscopic examination of Giemsa-stained thick and thin blood films. This method requires vigorously trained technicians to efficiently detect and classify the malaria parasite species such as *Plasmodium falciparum *(Pf) and *Plasmodium vivax *(Pv) for an appropriate drug administration. However, accurate classification of parasite species is difficult to achieve because of inherent technical limitations and human inconsistency. To improve performance of malaria parasite classification, many researchers have proposed automated malaria detection devices using digital image analysis. These image processing tools, however, focus on detection of parasites on thin blood films, which may not detect the existence of parasites due to the parasite scarcity on the thin blood film. The problem is aggravated with low parasitemia condition. Automated detection and classification of parasites on thick blood films, which contain more numbers of parasite per detection area, would address the previous limitation.

**Results:**

The prototype of an automatic malaria parasite identification system is equipped with mountable motorized units for controlling the movements of objective lens and microscope stage. This unit was tested for its precision to move objective lens (vertical movement, z-axis) and microscope stage (in x- and y-horizontal movements). The average precision of x-, y- and z-axes movements were 71.481 ± 7.266 μm, 40.009 ± 0.000 μm, and 7.540 ± 0.889 nm, respectively. Classification of parasites on 60 Giemsa-stained thick blood films (40 blood films containing infected red blood cells and 20 control blood films of normal red blood cells) was tested using the image analysis module. By comparing our results with the ones verified by trained malaria microscopists, the prototype detected parasite-positive and parasite-negative blood films at the rate of 95% and 68.5% accuracy, respectively. For classification performance, the thick blood films with Pv parasite was correctly classified with the success rate of 75% while the accuracy of Pf classification was 90%.

**Conclusions:**

This work presents an automatic device for both detection and classification of malaria parasite species on thick blood film. The system is based on digital image analysis and featured with motorized stage units, designed to easily be mounted on most conventional light microscopes used in the endemic areas. The constructed motorized module could control the movements of objective lens and microscope stage at high precision for effective acquisition of quality images for analysis. The analysis program could accurately classify parasite species, into Pf or Pv, based on distribution of chromatin size.

## Background

Malaria is a devastating endemic parasitic disease in tropical and subtropical areas that causes approximately 216 million infection cases with 655,000 deaths according to the World Malaria Report 2011 published by the World Health Organization (WHO) [1]. Rapid detection of infected cells and classification of parasite species can facilitate treatment of the disease with appropriate drugs and help control the spread of the disease. Routine methods for diagnosis of malaria parasites are by means of light microscopy and rapid diagnostic test kits [2]. Using light microscope is a standard approach to detect parasites, which involves preparation and microscopic examination of Giemsa-stained thick and thin films of blood from malaria patients. Furthermore, microscopic examination can be used for classification of species and also determination of the number of parasites. However, this process requires a vigorously trained technician to efficiently and accurately detect parasite and classify its species. With other factors such as some technical limitations and possible human inconsistency, the detection and classification can be further degraded.

To address the above issues, considerable research efforts were made to develop automatic malaria detection systems on thin blood smears [3-8]. For example, Tek et al. [3] employed *k*-nearest neighbour classifier to classify parasite species. The combination of various features were used including histogram, Hu moments, relative shape measurement, color auto-correlogram. Ross et al. [4] reported the use of feedforward backpropogation neural networks to detect malaria parasites on thin blood film; then classify the parasites into four species (*P. falciparum, P. vivax, P. ovale *and *P. malariae*). Seman et al. [5] used multilayer perceptron (MLP) network to classify the malaria parasites into three species (*P. falciparum, P. vivax *and *P. malariae*). They proposed six features (size of infected red blood cell (RBC) per size of normal RBC, shape of the parasite, numbers of the parasite's chromatin, numbers of parasite per RBC, texture of RBC and location of the chromatin) to be used in their classification protocol. Le et al., [6] proposed the detection system consisting of six stages: nucleated components detection, components detection, image decomposition, erythrocyte size estimation, leukocytes and malaria gametocytes classification, and parasitemia estimation. This system was tested on 200 smear samples and the reported average error is less than 1% comparing to manual evaluation. Diaz et al., [7] presented the method for both quantifying and classifying RBC on thin blood smears. During the classification process, this framework can also classify and differentiate the infection stages of *P. falciparum*. Savkare and Narote [8] presented an automatic system to distinguish the normal and infected RBC with *P. falciparum *or protozoan parasites. Nevertheless, thin blood film examination of malaria parasites is not practical especially with low level of parasitemia per detection area. Thus, thick blood film examination where layers of RBC are approximately 6-20 times thicker than the thin film can have better chances to detect parasites under low parasitemia condition.

There are number of automatic systems developed for counting of infected RBC on a thick blood film. Frean et al., described an automatic parasite counting system on thick blood film using image analysis techniques [9]. An extension of this work [10] presents a semi-automatic malaria counting system in thick blood film using open-source software. To our knowledge, there is no automatic image analysis for malaria species classification on thick blood film, which is very challenging even for an experienced microscopist because the early blood stage (trophozoites or "ring form") of all species appears identical and cannot be classified using merely the ring morphology. However, classification of species can be made possible based on the observation of several trophozoites and other parasite stages.

To make it possible for deploying an automatic system for detection and classification of malaria parasites in endemic areas, a thick blood film was chosen for our study. We implemented a prototype system for automatic detection and classification of malaria species on thick blood film. This system is composed of 1) image acquisition module and 2) malaria image analysis module specifically designed for handling thick blood film.

## Results

### Image acquisition module

#### Evaluation of image acquisition unit

**Figure 1:**
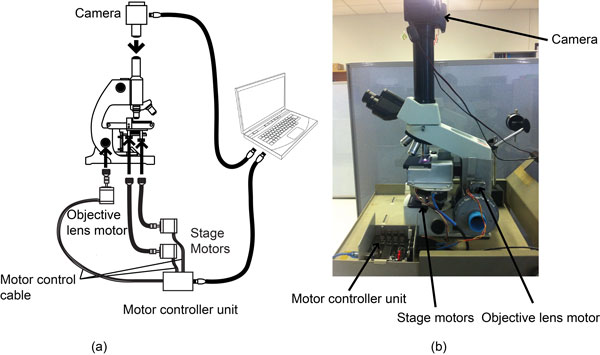
**Diagram and photo of motorized units for objective lens and stage movement**. (a) The schematic of automate microscope system. The system consists of motorized units, a motor controller unit and an eyepiece camera. There are three motorized units. Two of them control stage position called stage motorized unit and the objective lens motorized unit controls the objective lens position. An eyepiece camera is used to capture images and transfer to the image analysis module in a computer via USB connection. (b) Photo of assembly of all units.

**Figure 2:**
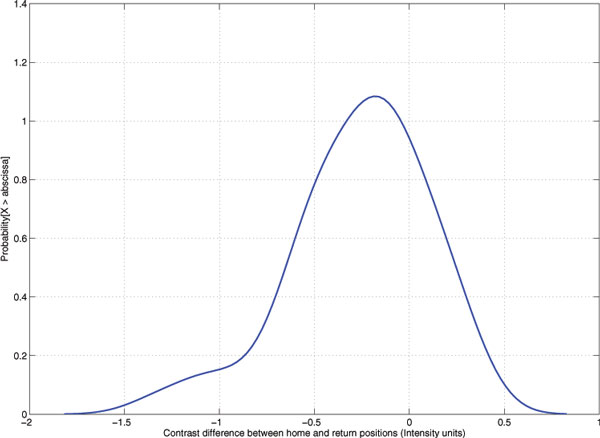
**Error distribution of contrast differences between the home position before and after movements**. The acquisition unit was set to move the microscope stage upward from the start point (set as home position) 500 steps, and then back to home position. The Root Mean Square (RMS) in equation 1 is utilized for computing image contrast for each movement. The error distribution of RMS differences between the home position and the position after upward and downward movement (return position) are plotted. The mean and standard deviation of the error are -0.263 and 0.357 respectively.

**Figure 3:**
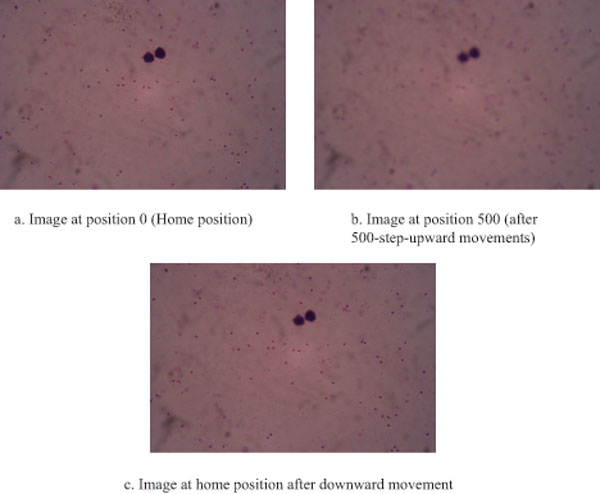
**Comparison of image quality at home position before forward and after backward movements**. The image of the start point (set as home position 3.a) was taken. Then, the objective lens is moved up 500 steps and then moved back to the home position. Images of the position 500 (3.b) and at home position after downward movement (3.c) were taken.

In order to collect images for analysis, a motorized unit controlling the precise movements of objective lens and microscope stage was designed (Figure 1). For objective lens movement test, the acquisition unit was set to move the objective lens up from a home position 500 steps, and then move back to the home position. The Root Mean Square (RMS) of image contrast was used to appraise this unit for each movement (Figure 1). The results showed that the image acquisition unit could accurately return to the home position with average error of -0.263 with standard deviation of 0.357 (Figure 2). The captured images at home position before and after the experiments are compared in Figure 3. The average precision of stage (x-y) and objective lens (z) movements were 71.481 ± 7.266 μm, 40.009 ± 0.000 μm, and 7.540 ± 0.889 nm respectively (Table 1).

**Table 1:** Precisions of all 3-directional movements.

Axis	Average Precision
z	7.540 ± 0.889 nm
x	71.481 ± 7.266 μm
y	40.009 ± 0.000 μm

For z-axis, the 10,000-step-forward movement was performed. The process was repeated 10 times. For x-axis and y-axis movements, 36 movements (18 forward movements and 18 backward movements) of each direction were performed. The distance was measured by a micrometer. For z-axis, the 10,000-step-forward movement allows micrometer to measure its average precision in nano-scale.

#### Image enhancement

**Figure 4:**
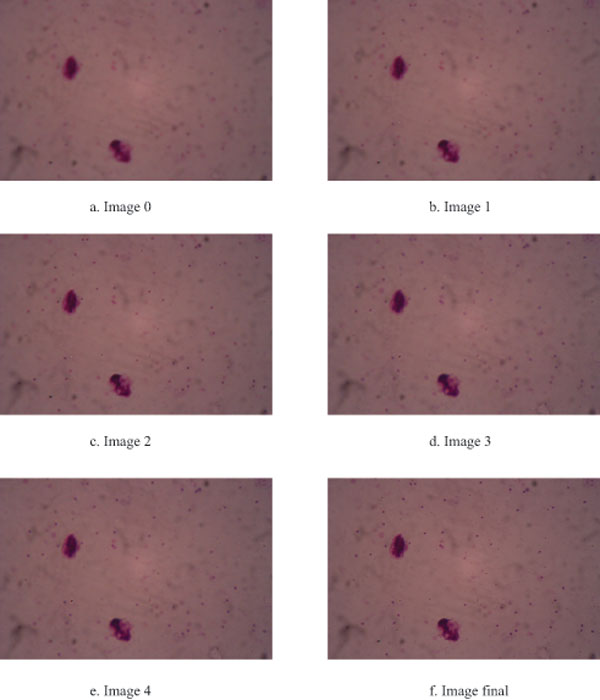
**Various depth of field images**. Images of each depth of field which have different in-focus area are shown in 4.a-4.e. The merging of in-focus information over various depths of field improves the quality of image (4.f).

Images from various depths of field were acquired using the z-movement control of the acquisition module. The acquired images were combined into one final image using extended depth of field technique so that the detail of each component can be accurately determined. Figure 4 illustrates images of each depth of field which have different in-focus areas. The merging of in-focus information over various depths of field improves the quality of image, labeled as 'Image final'.

### Image analysis module

#### Classification model based on chromatin size

**Figure 5:**
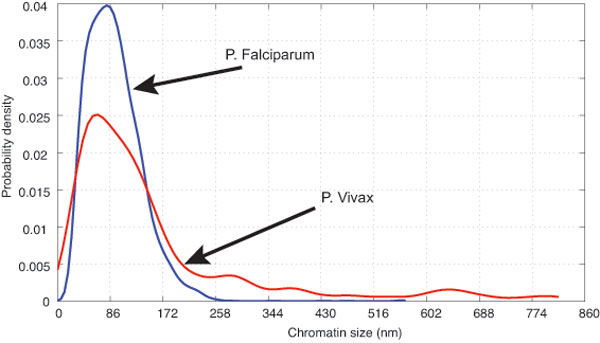
**Distribution of chromatin size in *P. falciparum *and *P. vivax *parasites**. The size of Pf's chromatin mainly distributes in the range of 30.1 - 258 nm, while Pv's chromatin size occupies in the wider range of 30.1 - 688 nm.

It is commonly known that Pv parasites tend to have larger size of chromatin than that of Pf parasites. However, it has never been quantified and hence, in this study, the chromatin size was quantified and used as the criteria to classify the species of malaria parasites. By acquiring the extended depth of field image, we measured the size of chromatin from 4,000 parasites and constructed the classification model. As shown in Figure 5, the chromatin size of both Pf and Pv ranges from 30.1 to 688 nm. In particular, the size of Pf's chromatin mainly varies in the range of 30.1 to 258 nm, while Pv's chromatin size has wider range of 30.1 to 688 nm. In addition, it was found that more than 20% of the Pv samples have the size of chromatin > 258 nm.

#### Detection and classification of malaria parasites

The prototype of automatic malaria identification system was evaluated by performing detection and classification of malaria parasites using 20 parasite-negative and 40 parasite-positive thick blood films. For *detection *test, the blood films containing either Pf or Pv parasites verified by trained microscopists were used. The results of identifying parasite-negative blood films from parasite-positive blood films (either Pf or Pv infection) are shown in Table 2. The detection accuracy of parasite-positive blood films was 95% while the success rate of correctly detecting parasite-negative blood films was 68.5%. For *classification *test, the thick blood films with Pv parasite was correctly classified with the success rate of 75% while the accuracy of Pf classification was 90% (Table 2).

**Table 2:** The evaluation of species classification performance

Thick blood films	No infection	Unknown object	Pv species	Pf species	Unknown species
Pv species	-	2/20	15/20	1/20	2/20
Pf species	-	-	2/20	18/20	-
No infection	14/20	6/20	-	-	-

The prototype of automatic malaria identification system was evaluated by performing detection and classification of malaria parasites using 20 parasite-negative and 40 parasite-positive thick blood films. For *detection *test, the blood films containing either Pf or Pv parasites verified by trained microscopists were used.

## Discussion

The goal of this study is to develop an automatic system for detection and classification of malaria parasite species in Giemsa-stained thick blood films. To date, a few automatic systems were developed to count numbers of parasites in thick blood films [9,10]. To our knowledge, however, there is no automatic system that could classify species of malaria parasites in thick blood films, especially for *P. falciparum *and *P. vivax *that are the two most prevalent species. One of the main reasons is that the morphology of both species are very similar when prepared as thick blood films and hence, it is very difficult to design an algorithm to distinguish one species from another. To overcome this difficulty, improvement of image acquisition method and finding of specific features -- that are unique in each parasite species, are needed.

The prototype of motorized image acquisition unit presented in this work accurately controls the movements of objective lens. The unit enabled us to fully automatically collect high quality images. In combination with the extended depth of field method, we could obtain in-focus images with all information of various depths of field for image analysis. However, this step is computationally time consuming. To control the computational time, the level of vertical movement was limited to 5 steps (2 upward steps, 2 downward steps and current step). The more we can minimize the computational time of this task, the more level of vertical movements can be performed; thus this leads to higher quality of in-focus images. The high image quality will directly improve the performance of detection and classification of malaria parasites.

Chromatin is a combination of DNA, RNA and proteins that condense DNA to fit within the nucleus of a cell. It exhibits solid color and its edge can be easily detected in Giemsa-stained blood film. Therefore, in this study, we use chromatin to detect and classify parasite species. On the other hand, cytoplasm edge is blended to the background and it is difficult to determine. Based on the results, there was a difference in chromatin size of Pf and Pv parasites. If the chromatin size is in the range of 64.5 - 258 nm, it will be classified as Pf. When the chromatin size falls in the range of 64.5 - 258 nm and the numbers of chromatin with the size greater than 258 nm is more than 20%, the parasite will be classified as Pv. Otherwise, the algorithm cannot conclusively classify the species and an intervention by a malaria technician performing standard microscopic observation is needed. The species classification performance could be improved by utilizing additional distinguishable features such as shape and color of the parasites.

Based on Table 2, the performance of distinguishing the parasite-positive films from parasite-negative films was at 95% with only 5% of parasite-positive films was identified as unknown object. One of the reasons of the mis-detection could be because of the low level of parasitemia in these blood films. To enhance the system performance to detect parasite cells when the level of parasitemia is low, other features of parasite cells can be used to further investigate each possible parasite region. If the region represents those parasite characteristics, it will be assigned as parasite-positive blood films even the number of parasite is very low. It is necessary that the patient is properly treated with antimalarial drug even though the parasite load is low. This is to prevent spread of the disease and reduce adaptation of parasite for living in human host. It was reported that people living in the endemic areas can also have low level of parasitemia without symptoms due to their adaptive immune response [11].

This prototype is designed to fully function automatically by using a motorized stage unit that controls the movements of objective lens for fine focus tuning and of microscope stage for facilitating acquisition of images in different regions. An operator only places a slide with Giemsa-stained thick blood films on the microscope stage and performs initial focusing. By starting the analysis program, the detection and classification processes will proceed automatically and report the species classification results on the screen. This device is user-friendly and useful for deployment in endemic areas to help health personnel in examination of malaria-infected blood samples for quick and appropriate treatment of malaria patients.

## Conclusions

In this study, we have developed an automatic system for detection and classification of malaria parasites in thick blood films. The prototype is equipped with motorized units to control stage and objective lens positions at high precision. These motorized units are designed to be easily mounted on most microscopes. Moreover, it employs image analysis unit which can accurately classify parasite into Pf or Pv species using distribution of chromatin size. The prototype can be deployed in endemic areas to assist local health personnel in malaria diagnosis.

## Methods

### Structure of the malaria detection and classification system

Structure of the system comprises two main components: (1) Image acquisition unit and (2) Image analysis module installed inside the processing unit. The image analysis module passes control commands to image acquisition unit and receives the captured images from a digital camera (Canon EOS 500D) installed on the trinocular head of a conventional laboratory microscope (Olympus CX31).

### Image acquisition unit

The goal of image acquisition is to acquire sufficiently decent quality images with crucial information for further manipulation such as interpretation of the image, i.e., malaria shape recognition. Especially for the biomedical images, the lack of crucial information may lead to misinterpretation. In order to get quality images, the image acquisition unit is designed to automatically controlling the movement of microscope stage in 3-directional planes. The x-axis and y-axis horizontal adjustments are used for repositioning a slide while the z-axis vertical adjustment is used for focusing. The vertical adjustment system operates the fine-focus knob of the microscope to vertically move the lens while the digital camera that is installed at the top of microscope capturing the 1000 × magnified images from various depths of field. All information from all depths of field is merged to get all in-focus parts. Information of only one depth of field is not enough for estimation of size and shape of a parasite and may directly affect the performance of species classification. The in-focus image is sent to image analysis module installed on a personal computer. The image analysis module detects and classifies malaria parasites on each field of the thick blood film. The analysis process is finished, when the pre-set number of fields is reached.

The image acquisition board consists of a micro-controller (PIC 16F877), a USB port connected to the processing unit and control ports connected to three motorized units. The z-axis motorized unit attached to the fine-tune knob of microscope operates using a 4-phase stepping motor. To achieve the nanometer scale, each step of stepping motor is divided into 16 sub-steps. For x- and y-axis, the motorized unit was designed that it moves to the adjacent microscope fields without overlapping with the previous field.

#### Motorization accuracy test

For z-axis movement test, the acquisition unit was set to move the objective lens upward from the start point (set as home position) 500 steps, and then move down 500 steps to home position. The Root Mean Square (RMS) [12] (equation 1) is utilized for computing image contrast for each movement.

(1) $$\text{RMS}=\sqrt{\frac{1}{MN}\overset{N-1}{\underset{i=0}{\sum }}\overset{M-1}{\underset{j=0}{\sum }}{\left(I_{i,j}-\bar{I}\right)}^{2}},\bar{I}\;\;\text{is the average intensity of pixels}$$

where *M *and *N *are the width and height of an image while *I_i,j _*is the intensity at *i*,*j *pixel positions.

For z-axis, a 10,000-step-upward movement was performed and the distance was measured by a micrometer. The process was repeated 10 times. For x-axis and y-axis movements, a set of 36 movements (18 forward and 18 backward movements) of each direction was performed and the distance was measured by a micrometer.

### Image analysis module

The image analysis module consists of five processes; image acquisition, pre-processing, image segmentation, feature extraction and classification. First, the blood film is placed on the microscope stage. The initial position and focus are set before the analysis processes are started. Motorized controller controls the movement of microscope stage by sending the movement command to each motorized unit that is attached to the microscope knob. The digital snapshot of one microscope field sample is automatically acquired with the digital camera and sent to pre-processing process. The function of pre-processing process is to control vertical movement so that the system is able to capture images in different depths of field. This benefits the system to improve the quality of image by merging in-focus information over a range of images to generate a single entirely in-focus image. The in-focus regions are identified based on their sharper details. Laplacian spatial filter [12] is used to detect edges for in-focus pixel positions of each depth of field image because of its high accuracy and high speed. We define *L(x,y) *as the Laplacian value at *x *and *y *positions, which ranges from 1 to *N *where *f(x,y) *denotes the value of the image, *X *and *Y *are the width and height of the image, respectively.

(2) $$L\left(x,y\right)=\underset{X}{\sum }\underset{Y}{\sum }\left\{\left|2f\left(x,y\right)-f\left(x-1,y\right)-f\left(x+1,y\right)\right|+\left|2f\left(x,y\right)-f\left(x,y-1\right)-f\left(x,y+1\right)\right|\right\}$$

Laplacian values of each depth of filed image are computed using above equation. The merging of in-focus information over various depths of field constructs an in-focus image for further analysis.

After pre-processing, the whole in-focus image is converted into HSV (Hue-Saturation-Value) color format. The value component of HSV image is employed for segmentation that is divided into three steps.

Step 1: Construct a histogram of value components and extract non-background objects (white blood cells, malaria parasites and possible Giemsa stain-derived artifacts). These are extracted using adaptive threshold [13,14] found according to information of the histogram.

Step 2: After discarding the background, the image is divided into small windows of 300 by 300 pixels for efficient use of resources in searching process. The connected regions are then searched and each region was labelled with an identification value for future reference.

Step 3: In each window, malaria parasites are then distinguished from white blood cells according to their difference in sizes (white blood cells are larger than malaria parasites).

The above processes (steps 2-3) are repeated until all the parasites are discovered and labelled.

Next, the labelled regions that may contain the parasites are further processed. As the hue values represent different physical components of the parasites, the hue histogram of HSV image is constructed. The chromatin size represented by the number of red and magenta pixels in the hue histogram of each region are used for distinguishing chromatin from background in the classification process.

Using the extracted feature (chromatin size), malaria parasites are classified into two species, *P. falciparum *(Pf) and *P. vivax *(Pv) based on the difference of chromatin size of which the Pf parasites have a smaller size of chromatin than that of the Pv. The numbers of Pf and Pv cells in all microscopic fields are counted and recorded. For those blood films where classification is not possible, they will be designated as infected with unknown species, and the system will alert the controlling technician that the sample contains malaria parasites, but the species classification needs to be confirmed by standard microscopic observation method.

The performances of segmentation process and classification process were examined. Segmentation process aims to segment interested objects, which are white blood cells and parasites, from the background. A good segmentation process should also be able to segment region of interests in various lighting conditions. After that, each segmented parasite object is then sent to classification process for species classification. The process must be able to classify the parasite species correctly.

#### Segmentation process

To test the performance of segmentation process, over 360 images of thick blood films, containing Pf or Pv parasites, prepared in various field environments were used. Each image at the resolution of 928 × 616 with 24-bit depth was converted to HSV values and the histogram was generated by V values. The histogram range of background was eliminated while the histogram range of interested objects was preserved using adaptive thresholding [14] depending on the characteristic of individual image. By using adaptive histogram method, the process was able to correctly extract the interested objects (white blood cells, malaria parasites and possible Giemsa stain-derived artefacts) from the background. After the interested object regions were obtained, the malaria parasite regions were distinguished from the white blood cells and artefact regions according to the size. Each malaria parasite region was further analyzed in feature extraction and classification processes to classify parasite species.

#### Classification process

Using the extracted feature (chromatin size), malaria parasites were then classified into two species, *P. falciparum *(Pf) and *P. vivax *(Pv) the two most prevalent species. Based on our observation, Pf parasites have smaller size of chromatin than those of Pv. To verify this argument, chromatin size of a total 4,000 samples of both parasite species were investigated.

In our designed classification process, the parasite cells in all microscope fields were recorded and analyzed for the distribution of chromatin size. The decision process was then performed by evaluating the distribution of chromatin size as in the following criteria:

• number of parasite = 0: classified as no infection

• chromatin size < 64.5 nm: classified as unknown object

• 64.5 nm < chromatin size < 258 nm: classified as Pf parasite

• 64.5 nm < chromatin size < 688 nm, and the amount of chromatin size of > 258 nm greater than 20 percents: classified as Pv parasite

Otherwise, it was designated as infection with unknown species since the process cannot classify the species of parasite. When the blood films are classified as artefact and infection with unknown species, it will alert technicians to perform further manual investigation.

Using classification module above, the captured uncompressed images at a resolution of 928 × 616 with 24-bit depth, were processed to get for in-focus image from 5 depths of field (2 upward steps, 2 downward steps and current step). Then, the in-focus image was passed to the pre-processing process, segmentation process, feature extraction process and classification process. All processes were repeated until certain numbers of microscope fields were analyzed.

## Competing interests

The authors declare that they have no competing interests.

## Authors' contributions

SC and ST conceived the original idea of this research. SK and AI implemented the image processing tools. MP and AI designed and implemented the image acquisition unit. SK, AI and CU designed and conducted the experiments. SK, CU, AI and ST wrote the manuscript. All authors read and approved the final manuscript.
